# Norepinephrine Dose and Pulse Oximetry Agreement During Critical Illness: A Prospective Repeated-Measures Study

**DOI:** 10.3390/medicina62091687

**Published:** 2026-09-03

**Authors:** Mahmut Yilmaz, Mete Erdemir, Mehmet Celal Ozturk, Rahime Aydin Kayali, Burak Emre Gilik, Mehmet Cuhadar, Huseyin Ozkarakas, Gurhan Taskin, Levent Yamanel

**Affiliations:** 1 Department of Intensive Care, Gulhane Training and Research Hospital, Ankara 06010, Türkiye; 2 Department of Intensive Care, Izmir City Hospital, Sevket Ince Neighborhood, 2148/11. Street No:1/11, Izmir 35540, Türkiye; 3 Department of Intensive Care, Mengucek Gazi Training and Research Hospital, Erzincan 24100, Türkiye

**Keywords:** pulse oximetry, arterial oxygen saturation, norepinephrine, critically ill patients, measurement agreement, intensive care unit

## Abstract

*Background and Objectives*: Pulse oximetry is essential in ICUs but may be less accurate when peripheral perfusion is impaired. Norepinephrine, the primary vasopressor for shock, may exacerbate vasoconstriction and microcirculatory alterations, increasing discrepancy between pulse oximetry saturation (SpO_2_) and arterial oxygen saturation (SaO_2_). This study aimed to evaluate whether norepinephrine dose is associated with disagreement between SpO_2_ and SaO_2_, with particular attention to measurement variability and large discrepancies. *Materials and Methods*: We analyzed 1050 paired SpO_2_–SaO_2_ measurements from 196 adult ICU patients receiving norepinephrine. Dose was weight-adjusted (µg/kg/min). Based on the clinically relevant thresholds defined in the 2021 Surviving Sepsis Campaign guidelines for initiating vasopressin, doses were categorized as low (≤0.25 µg/kg/min), moderate (0.25–0.50 µg/kg/min), or high (≥0.50 µg/kg/min). A large discrepancy was defined as |SpO_2_ − SaO_2_| ≥ 5%. A generalized estimating equation (GEE) model was applied to account for repeated measurements within patients. *Results*: Overall mean bias was 0.91 ± 3.58%. In the primary GEE analysis accounting for repeated measurements within patients, norepinephrine dose was not significantly associated with large SpO_2_–SaO_2_ discrepancies when analyzed as a continuous variable (adjusted OR 0.964, 95% CI 0.916–1.014, *p* = 0.158). Categorical analyses showed a non-monotonic pattern, with only the comparison between the moderate- and high-dose groups reaching statistical significance and with wide confidence intervals limiting the precision of this finding. Unadjusted descriptive analyses suggested greater measurement variability across dose ranges, which was largely attributable to within-patient clustering. ROC analysis demonstrated limited discriminatory ability for norepinephrine dose alone (AUC 0.60). *Conclusions*: After accounting for repeated measurements and relevant clinical covariates, norepinephrine dose did not demonstrate a consistent dose-dependent association with large SpO_2_–SaO_2_ discrepancies. Although large SpO_2_–SaO_2_ discrepancies may occur in critically ill patients receiving norepinephrine, norepinephrine infusion rate alone should not be used as a surrogate marker of pulse oximetry reliability.

## 1. Introduction

Pulse oximetry is a widely used, noninvasive monitoring tool that provides continuous estimates of arterial oxygen saturation (SpO_2_) and plays a central role in oxygen titration and clinical decision-making in critically ill patients. Its ease of use and real-time availability have led to near-universal adoption in intensive care units (ICUs). However, SpO_2_ represents an indirect optical estimate and may diverge from arterial oxygen saturation measured by co-oximetry (SaO_2_), particularly under conditions that impair signal acquisition or alter peripheral perfusion [1].

Several physiological and technical factors are known to compromise pulse oximetry accuracy, including hypotension, hypothermia, motion artifact, dyshemoglobinemia, severe anemia, and reduced peripheral perfusion. These limitations are particularly relevant in ICU populations, where the use of vasopressors, circulatory shock, and microvascular dysfunction are common. Regulatory authorities and international standards bodies have emphasized that pulse oximeter performance is context-dependent and that accuracy may deteriorate under conditions of poor circulation or vasoconstriction [2,3,4]. Accordingly, the U.S. Food and Drug Administration (FDA) and the International Organization for Standardization (ISO 80601-2-61) acknowledge that device accuracy demonstrated in controlled testing may not fully reflect real-world ICU performance [3,5].

Norepinephrine is the first-line vasopressor for many forms of shock and is routinely administered to restore mean arterial pressure in critically ill patients. While norepinephrine improves macrocirculatory parameters, it may exacerbate peripheral vasoconstriction and alter microcirculatory flow, potentially impairing pulse oximetry signal fidelity. Experimental and clinical data suggest that reduced peripheral perfusion and vasopressor exposure are associated with greater discrepancies between SpO_2_ and SaO_2_ [4]. Despite this biologically plausible mechanism, the extent to which norepinephrine dose influences measurement agreement remains insufficiently characterized.

Importantly, mean bias alone may not adequately reflect measurement performance, as small average differences may coexist with substantial variability and clinically relevant outliers. Therefore, evaluating both the magnitude and distribution of discrepancies is essential for understanding the clinical reliability of pulse oximetry in critically ill patients.

This study aimed to investigate whether norepinephrine dose is independently associated with SpO_2_–SaO_2_ disagreement in ICU patients using a repeated-measures analytical approach that accounts for within-patient correlation, with particular focus on measurement variability and the impact of repeated measurements.

## 2. Methods

### 2.1. Study Design and Population

This prospective observational study was conducted in the intensive care unit of Gülhane Training and Research Hospital. Ethical approval for the study was obtained from the local ethics committee, and the study was conducted in accordance with the Declaration of Helsinki. Written informed consent was obtained from patients or their legal representatives, as required by local regulations.

Adult patients admitted to the intensive care unit between 1 July 2022, and 1 July 2023, who underwent simultaneous arterial blood gas analysis and pulse oximetry monitoring during norepinephrine infusion were prospectively enrolled. A total of 1050 paired SpO_2_–SaO_2_ measurements obtained from 196 patients were included in the final analysis.

Measurements were obtained as part of routine clinical practice, either at scheduled intervals (every 8–12 h) or when clinically indicated. Additional arterial blood gas samples were drawn at the discretion of the treating physician in response to: (i) a sudden decline in SpO_2_ (≥5% decrease from baseline), (ii) changes in ventilatory settings (e.g., increase in FiO_2_ or PEEP), (iii) hemodynamic instability (e.g., hypotension requiring fluid or vasopressor escalation), (iv) suspected hypoxemia despite acceptable SpO_2_ readings, or (v) routine post-procedural assessments. The median number of measurements per patient was 5 (interquartile range 3–8). No fixed-time-interval protocol was imposed, reflecting real-world ICU practice. Measurements were obtained at the time of arterial blood gas sampling.

**Figure 1:**
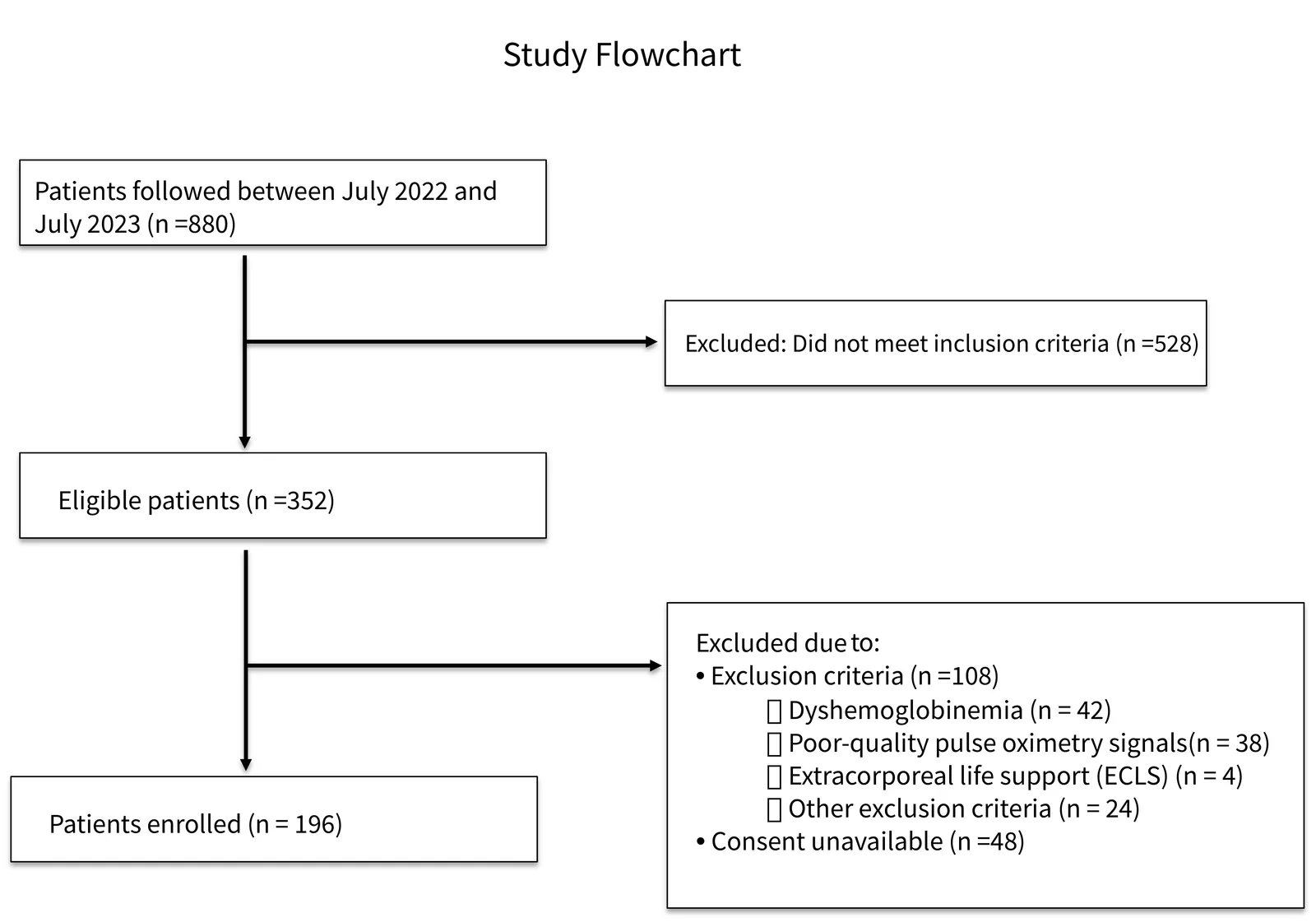
Study flowchart.

For the analysis of SpO_2_–SaO_2_ agreement, measurements obtained during concurrent administration of any additional vasoactive agent (e.g., vasopressin, dobutamine, epinephrine) were excluded. However, measurements from the same patient at other times, when only norepinephrine was administered, were retained. Thus, our analysis included all measurements obtained during periods of norepinephrine monotherapy. Patients were excluded entirely from the study if they had documented dyshemoglobinemia, exhibited poor-quality pulse oximetry signals (including severe motion artifact, unstable waveform, or probe malfunction) at any time during the study period, were supported with extracorporeal life support, or if informed consent could not be obtained (Figure 1). There were no missing data for any of the study variables, as all parameters were prospectively recorded completely. Consequently, no patients were excluded due to incomplete data, and all analyses were performed on a complete-case basis.

Demographic characteristics, disease severity (Acute Physiology and Chronic Health Evaluation II—APACHE II score, calculated using the worst physiological values during the first 24 h of ICU admission), primary clinical diagnosis, use of mechanical ventilation, presence of sepsis or septic shock, lactate levels, fraction of inspired oxygen (FiO_2_) as recorded at the time of measurement, and norepinephrine dose at the time of measurement were recorded. All covariates, including FiO_2_, lactate, and norepinephrine dose, were recorded at the time of each arterial blood gas sampling, ensuring temporal alignment with the paired SpO_2_–SaO_2_ measurement. Sepsis, septic shock, and mechanical ventilation status were recorded at ICU admission, whereas lactate, FiO_2_, and norepinephrine dose reflect the physiological status at the time of each paired measurement.

Pulse oximetry oxygen saturation was continuously monitored using standard bedside ICU monitors (Philips IntelliVue MP20, Philips Medical Systems, Böblingen, Germany) equipped with a Masimo Technology Adult SpO_2_ Sensor (Masimo Corp., Irvine, CA, USA). The sensor was applied to the index finger, and SpO_2_ values were recorded after signal stabilization and confirmation of an adequate plethysmographic waveform. The same finger was not used consistently across all measurements, as sensor placement was determined by the clinical team in accordance with routine clinical practice and was not systematically documented. This variability in sensor placement may have contributed to differences in signal quality and represents a potential source of measurement variability. SpO_2_ values were recorded at the time of arterial blood sampling. Although the SpO_2_ reading was obtained immediately at the moment of sampling, no electronically recorded time interval between the two measurements was available; therefore, the measurements are considered clinically simultaneous rather than confirmed by time-stamped electronic data.

Arterial oxygen saturation (SaO_2_) was measured directly by co-oximetry from arterial blood samples obtained using dry lithium heparinized syringes (BERİKA Blood Gas Sampling Set, Konya, Türkiye) in accordance with standard clinical practice. Blood gas analyses were performed immediately after sampling using an ABL 800 FLEX blood gas analyzer (Radiometer Medical, Copenhagen, Denmark), which measures oxygen saturation directly by co-oximetry rather than calculating it from derived parameters.

Norepinephrine was administered as norepinephrine tartrate. All infusion rates were converted to norepinephrine base equivalents using the standard conversion factor (1 mg of norepinephrine tartrate = 0.68 mg of norepinephrine base) and expressed in µg/kg/min to ensure comparability across patients. All doses were weight-adjusted using actual body weight measured at ICU admission.

For categorical analyses, norepinephrine exposure was stratified into three dose groups based on weight-adjusted infusion rate:

- Low dose: ≤0.25 µg/kg/min;
- Moderate dose: 0.25–0.50 µg/kg/min;
- High dose: ≥0.50 µg/kg/min.

These thresholds were chosen based on the clinically relevant cutoffs defined in the 2021 Surviving Sepsis Campaign guidelines for vasopressin initiation [6]; however, they have not been validated specifically for pulse-oximeter performance and are used here for descriptive categorization only. The norepinephrine dose was additionally analyzed as a continuous variable to avoid information loss associated with categorization.

Both signed and absolute differences between pulse oximetry and arterial oxygen saturation were analyzed to provide a comprehensive assessment of pulse oximetry performance across varying norepinephrine exposure levels.

The signed saturation difference (SpO_2_ − SaO_2_) was calculated and used to assess the direction of measurement bias (systematic overestimation or underestimation). The absolute saturation difference, |SpO_2_ − SaO_2_|, was calculated to quantify the magnitude of measurement error and reflect overall agreement and reliability regardless of bias direction.

Signed differences were reserved for bias assessment, whereas absolute differences were used to evaluate prespecified large discrepancy across dose groups, as opposite directional biases may cancel out when using signed differences alone.

A prespecified threshold of ≥5% was selected to define a large discrepancy between SpO_2_ and SaO_2_, as it exceeds the FDA-recognized accuracy root mean square (ARMS) of approximately 3% observed in healthy volunteers under controlled conditions [7]. This threshold has been used in recent ICU studies to identify large discrepancies [2,3], and is consistent with the performance standards outlined in ISO 80601-2-61 [5]. Sensitivity analyses were repeated using a ≥4% threshold, based on the 2-SD rationale under the assumption of normality for measurement differences [8]. Measurements meeting this criterion were classified as high error.

Bland–Altman analysis was performed solely as a descriptive, unadjusted exploratory visualization to illustrate the pattern of agreement and measurement dispersion across norepinephrine dose groups. Limits of agreement were calculated as mean bias ± 1.96 × standard deviation of the differences. Because this descriptive analysis does not account for repeated measurements, it is not considered a primary or confirmatory analysis; all formal inferences regarding dose-effect relationships are based on the GEE model. Proportional bias was assessed by linear regression of the SpO_2_–SaO_2_ difference against the mean of the two measurements [8]. A sensitivity analysis restricted to the first measurement per patient was performed and presented in the Supplementary Materials to explore the impact of data reduction on descriptive agreement patterns. For exploratory descriptive purposes only, we categorized the magnitude of SpO_2_–SaO_2_ discrepancies into four fixed absolute difference thresholds: <2%, 2–5%, 5–10%, and >10%. These thresholds were chosen pragmatically to visualize the distribution of measurement errors (e.g., <2% approximates excellent agreement, 2–5% captures moderate deviations, and ≥5% thresholds represent larger discrepancies that may warrant clinical attention). However, unlike classical error grid analyses that incorporate saturation-dependent clinical risk [9], this classification uses fixed absolute thresholds independent of the underlying saturation level and is intended solely to illustrate measurement dispersion, not to stratify clinical risk. Because this analysis does not account for repeated measurements, it was treated as purely exploratory and relegated to the Supplementary Materials; no formal trend testing was applied or inferred.

This study followed the Strengthening the Reporting of Observational Studies in Epidemiology (STROBE) reporting guideline for observational studies [10]. The completed STROBE checklist is available as Supplementary Table S1.

### 2.2. Statistical Analysis

Statistical analyses were performed using SPSS Statistics version 22.0 (IBM Corp., Armonk, NY, USA). Continuous variables were assessed for distributional characteristics using graphical methods and formal normality testing. Given the non-normal distribution of primary outcome variables, continuous data are presented as median (interquartile range) or mean ± standard deviation, as appropriate.

Paired comparisons between SpO_2_ and SaO_2_ were performed using the Wilcoxon signed-rank test. Differences across norepinephrine dose groups were assessed using the Kruskal–Wallis test, followed by post hoc Mann–Whitney U tests with Bonferroni correction, where applicable.

Effect sizes for Mann–Whitney U tests were calculated using *r* = *Z*/√*N*. Effect size for Kruskal–Wallis tests was calculated using epsilon-squared (*ε*^2^ = [*H* − *k* + 1]/[*N* − *k*]), where *H* denotes the Kruskal–Wallis statistic, *k* the number of groups, and *N* the total sample size.

Correlations between weight-adjusted norepinephrine dose and saturation discrepancies were evaluated using Spearman’s rank correlation coefficient.

As an exploratory secondary analysis, ROC curve analysis was performed to evaluate the discriminatory ability of weight-adjusted norepinephrine dose for identifying large SpO_2_–SaO_2_ discrepancies (≥5%). The AUC was reported with its 95% confidence interval. Cutoff values were explored using Youden’s index, but given the exploratory nature of this univariate analysis and the anticipated weak discrimination, no clinical threshold derivation was intended.

To account for within-patient correlation arising from repeated measurements, generalized estimating equations (GEE) with robust standard errors were used as the primary inferential approach. The continuous-dose GEE model was defined a priori as the primary exposure analysis, because it tests for a linear dose–response relationship without imposing arbitrary cutoffs and avoids the information loss inherent in categorization. The categorical analysis was retained as a secondary descriptive approach to allow clinical interpretation and comparison with prior studies. The binary dependent variable was defined as the presence of a large discrepancy (|SpO_2_ − SaO_2_| ≥ 5%), chosen to identify factors associated with clinically problematic errors rather than the continuous measurement magnitude. Covariates were selected a priori based on clinical plausibility and included norepinephrine dose group, FiO_2_, lactate, sepsis, septic shock, mechanical ventilation, and age. Sepsis and septic shock were included as separate covariates because they represent distinct clinical severity levels. Age was included as a covariate regardless of its statistical significance in order to adjust for its potential confounding effect, which was determined on clinical grounds. Multicollinearity was evaluated using variance inflation factors (VIF), with all values below 2 indicating no significant collinearity. An exchangeable working correlation structure was selected based on both clinical plausibility and model fit. The exchangeable structure assumes equal correlation among all measurements within the same patient, which was considered clinically reasonable given that all measurements were obtained during norepinephrine monotherapy under irregular time intervals. Model fit was compared using the Quasi-likelihood under the Independence Model Criterion (QIC), where the exchangeable structure demonstrated superior fit (QIC = 845.016; QICC = 799.663) compared with AR(1) (QIC = 880.715; QICC = 828.204). The unstructured correlation structure failed to converge due to variable cluster sizes (median 5, IQR 3–8). Sensitivity analyses using the AR(1) structure yielded consistent effect estimates. Hemoglobin, body temperature, and perfusion index were not included as covariates because they were not systematically recorded in the dataset. A two-sided *p*-value < 0.05 was considered statistically significant. APACHE II score was not included in the adjusted model because it reflects baseline disease severity at ICU admission rather than the physiological status at the time of each paired measurement, which was the focus of our primary analysis. Adding APACHE II to the model in a sensitivity analysis did not materially change the effect estimates.

There were no missing data for any of the study variables, as all parameters were prospectively recorded completely. Consequently, no patients were excluded due to incomplete data, and all analyses were performed on a complete-case basis. No imputation procedures were performed. Sample size was determined pragmatically by consecutive enrollment of eligible patients during the 1-year study period.

## 3. Results

A total of 1050 paired SpO_2_–SaO_2_ measurements obtained from 196 critically ill adult patients were included in the analysis (patient flow is shown in Figure 1).

The study cohort was characterized by advanced age and high disease severity, with a median age of 72 years (interquartile range, IQR, 59–78) and the median APACHE II score of 28 (IQR 23–37) (Table 1). The median number of measurements per patient was 5 (IQR 3–8).

The signed difference between pulse oximetry and arterial oxygen saturation (SpO_2_–SaO_2_) showed a mean of 0.91 ± 3.58% and a median of 0.60% (IQR −1.20 to 2.60), indicating a small overall bias toward higher SpO_2_ values. The absolute SpO_2_–SaO_2_ difference demonstrated a median of 2.00% (IQR 1.00–3.60) and a mean of 2.68 ± 2.53%, reflecting substantial variability across measurements (Supplementary Table S2). This dissociation between low mean bias and high variability suggests that average agreement metrics alone may underestimate clinically relevant measurement error. Occult hypoxemia, defined as SpO_2_ ≥ 92% with a concurrent SaO_2_ < 88%, was observed in 36 of 1050 measurements (3.4%).

In the primary GEE analysis accounting for intra-patient correlation, norepinephrine dose did not demonstrate a consistent dose–response relationship with large SpO_2_–SaO_2_ discrepancies (defined as |SpO_2_ − SaO_2_| ≥ 5%). Compared with the high-dose group (≥0.50 µg/kg/min), the adjusted odds ratios for the low-dose (≤0.25 µg/kg/min) and moderate-dose (0.25–0.50 µg/kg/min) groups were 1.80 (95% CI 0.74–4.35, *p* = 0.193) and 3.49 (95% CI 1.17–10.44, *p* = 0.025), respectively. While the moderate-dose comparison reached statistical significance, the wide confidence intervals reflect limited precision, and the overall categorical pattern does not support a consistent monotonic dose–response relationship. When analyzed as a continuous variable (per 0.1 µg/kg/min increment), norepinephrine dose was not statistically significantly associated with large SpO_2_–SaO_2_ discrepancies. The adjusted odds ratio was 0.964 (95% CI 0.916–1.014, *p* = 0.158). The quadratic term for norepinephrine dose was not significant (*p* = 0.452), indicating no evidence of nonlinearity. In a prespecified sensitivity analysis using a ≥4% threshold to define discrepancy, the results were consistent with the primary analysis. When analyzed as a continuous variable (per 0.1 µg/kg/min increment), norepinephrine dose remained not statistically significantly associated with the outcome (adjusted OR = 0.979, 95% CI 0.938–1.023, *p* = 0.347). In an exploratory analysis, we examined whether the association between norepinephrine dose and large discrepancy varied across clinically relevant SpO_2_ saturation strata (<90%, 90–94%, ≥95%). The interaction term between norepinephrine dose and saturation strata was not statistically significant (Wald χ^2^ = 0.813, df = 2, *p* = 0.666 for the primary ≥ 5% threshold; and Wald χ^2^ = 2.853, df = 2, *p* = 0.240 for the ≥4% threshold), indicating that the effect of norepinephrine dose did not differ significantly across saturation levels. These results suggest that the lack of association between norepinephrine dose and pulse oximetry error was consistent across the observed saturation range. The results of the multivariable GEE model are presented in Table 2.

**Figure 2:**
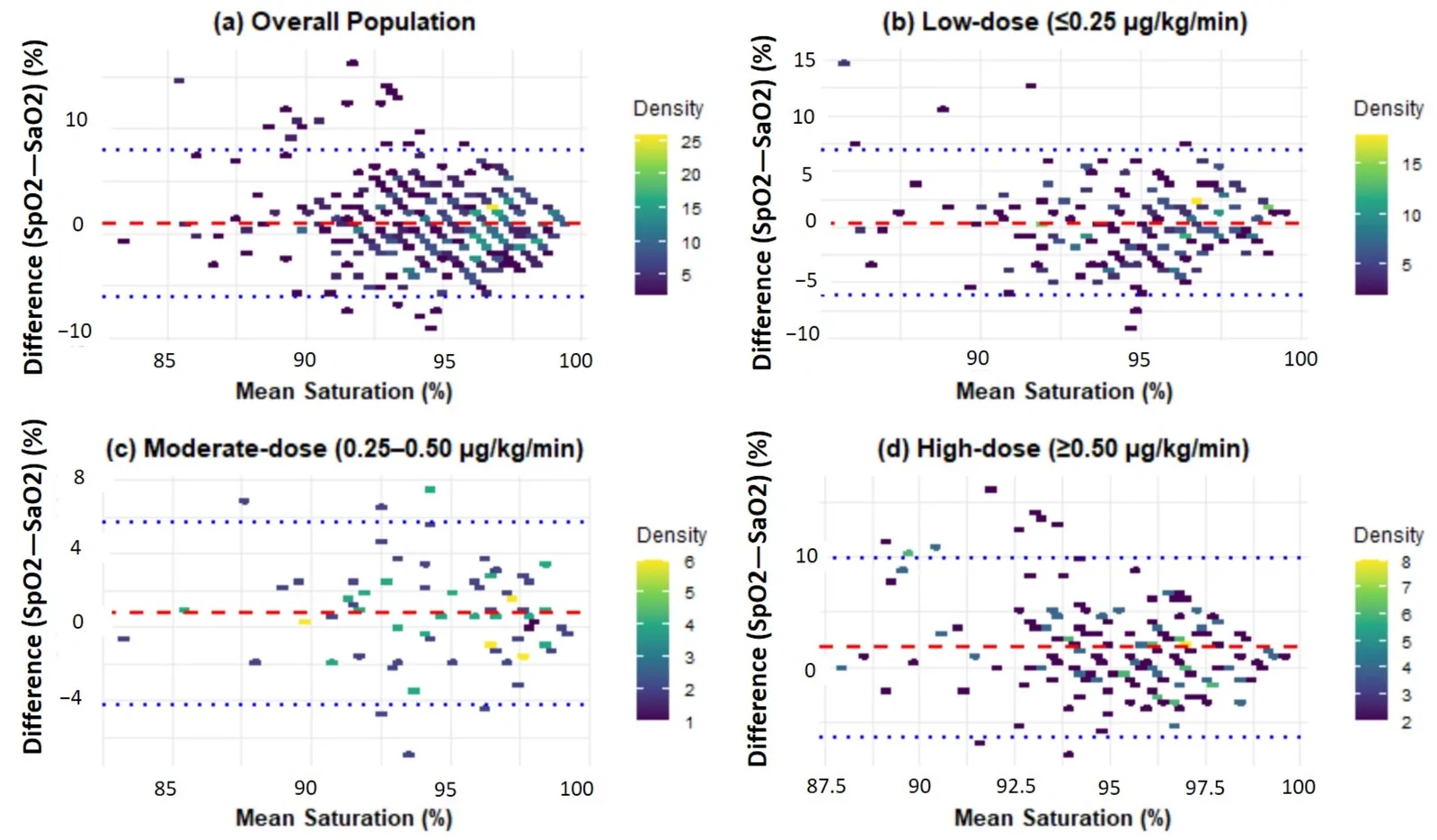
Bland–Altman plots of SpO_2_ versus SaO_2_ stratified by norepinephrine dose group. The red dashed line represents the mean bias (mean difference between SpO_2_ and SaO_2_), whereas the blue dotted lines represent the 95% limits of agreement (mean bias ± 1.96 SD).

In unadjusted, measurement-level descriptive analyses (which do not account for repeated measurements), paired analysis revealed a systematic discrepancy between pulse oximetry and arterial blood gas measurements. Median SaO_2_ was 95.4% (IQR 92.7–97.0), whereas median SpO_2_ was 96.0% (IQR 93.75–98.0), with the difference being statistically significant (*p* < 0.001) (Figure 2). Overall, pulse oximetry overestimated arterial oxygen saturation in 57.0% of measurements. In these unadjusted descriptive analyses, both the signed and absolute discrepancies appeared to vary significantly across norepinephrine dose groups (Supplementary Table S3).

Data are presented as median (interquartile range). Comparisons across norepinephrine dose groups were performed using the Kruskal–Wallis test. Post-hoc pairwise comparisons were conducted using Mann–Whitney U tests with Bonferroni correction (adjusted significance level α = 0.017). Signed differences reflect directional bias (SpO_2_ − SaO_2_), whereas absolute differences reflect the magnitude of measurement discordance irrespective of direction.

Panels show the overall cohort (a, *n* = 1050) and dose groups: low (≤0.25 µg/kg/min) (b, *n* = 510), moderate (0.25–0.50 µg/kg/min) (c, *n* = 178), and high (≥0.50 µg/kg/min) (d, *n* = 362). Color intensity reflects data density to reduce overplotting. The red dashed line indicates the mean bias, and the blue dotted lines show 95% limits of agreement. These plots are exploratory, unadjusted, and do not account for repeated measurements within patients; they should not be interpreted as confirmatory evidence of a dose-dependent effect. The primary inferential analysis is the GEE model presented in Table 2. 

Comparison of pulse oximetry saturation (SpO_2_) and arterial oxygen saturation (SaO_2_) according to norepinephrine dose group: low dose (≤0.25 µg/kg/min, *n* = 510, blue circles), moderate dose (0.25–0.50 µg/kg/min, *n* = 178, green circles), and high dose (≥0.50 µg/kg/min, *n* = 362, red circles). The black dashed line represents the line of identity (SpO_2_ = SaO_2_). Points above the line indicate SpO_2_ overestimation; points below indicate underestimation.

Descriptive Bland–Altman sensitivity analyses restricted to the first measurement per patient demonstrated similar patterns of increased variability at higher norepinephrine doses (Supplementary Figure S1; Supplementary Table S4). However, these descriptive findings are unadjusted for repeated measurements and are secondary to the primary GEE analysis.

In these unadjusted descriptive analyses, post-hoc comparisons revealed a stepwise increase in absolute discrepancy, most pronounced in the high-dose group. For signed differences, statistical significance persisted only between the low- and high-dose groups, whereas adjacent dose comparisons were no longer significant after correction for multiple testing. Spearman’s rank correlation coefficient showed a very weak and clinically negligible correlation between weight-adjusted norepinephrine dose and the absolute SpO_2_–SaO_2_ difference (ρ = 0.069, *p* = 0.025).

Large discrepancies, defined as an absolute SpO_2_–SaO_2_ difference of at least 5%, occurred in 13.9% of all paired measurements (Supplementary Table S2). The prevalence of large discrepancies differed significantly across norepinephrine dose groups (*p* < 0.001), occurring in 10.6%, 7.9%, and 21.5% of measurements in the low-, moderate-, and high-dose groups, respectively. The proportion of measurements with large discrepancies appeared higher in the high-dose group in this descriptive analysis.

In the GEE model, higher FiO_2_ remained independently associated with a lower likelihood of large discrepancies. For each 1 percentage point increase in FiO_2_, the adjusted odds ratio was 0.976 (95% CI 0.964–0.989, *p* < 0.001), corresponding to an approximately 24% reduction in the odds of a large discrepancy for a 10 percentage point absolute increase in FiO_2_ (OR 0.76, 95% CI 0.65–0.89). Other covariates, including age (analyzed as a continuous variable), were not statistically significantly associated with the outcome (Table 2).

In an exploratory sensitivity analysis, the discriminatory ability of norepinephrine dose for identifying large discrepancies was assessed using receiver operating characteristic (ROC) analysis. Norepinephrine dose showed only limited discriminatory ability (AUC = 0.60, 95% CI 0.55–0.65). The ROC curve is provided in Supplementary Figure S2. This modest AUC is consistent with, and further supports, the primary GEE finding that norepinephrine dose alone does not reliably distinguish patients who experience large measurement discrepancies. Although Youden’s index yielded an exploratory cutoff value (0.59 µg/kg/min), the overall weak discriminatory performance (AUC 0.60) indicates that no clinically useful cutoff can be derived from this single-variable analysis. Therefore, we do not recommend using a specific norepinephrine dose threshold in clinical decision-making regarding pulse oximetry reliability.

**Figure 3:**
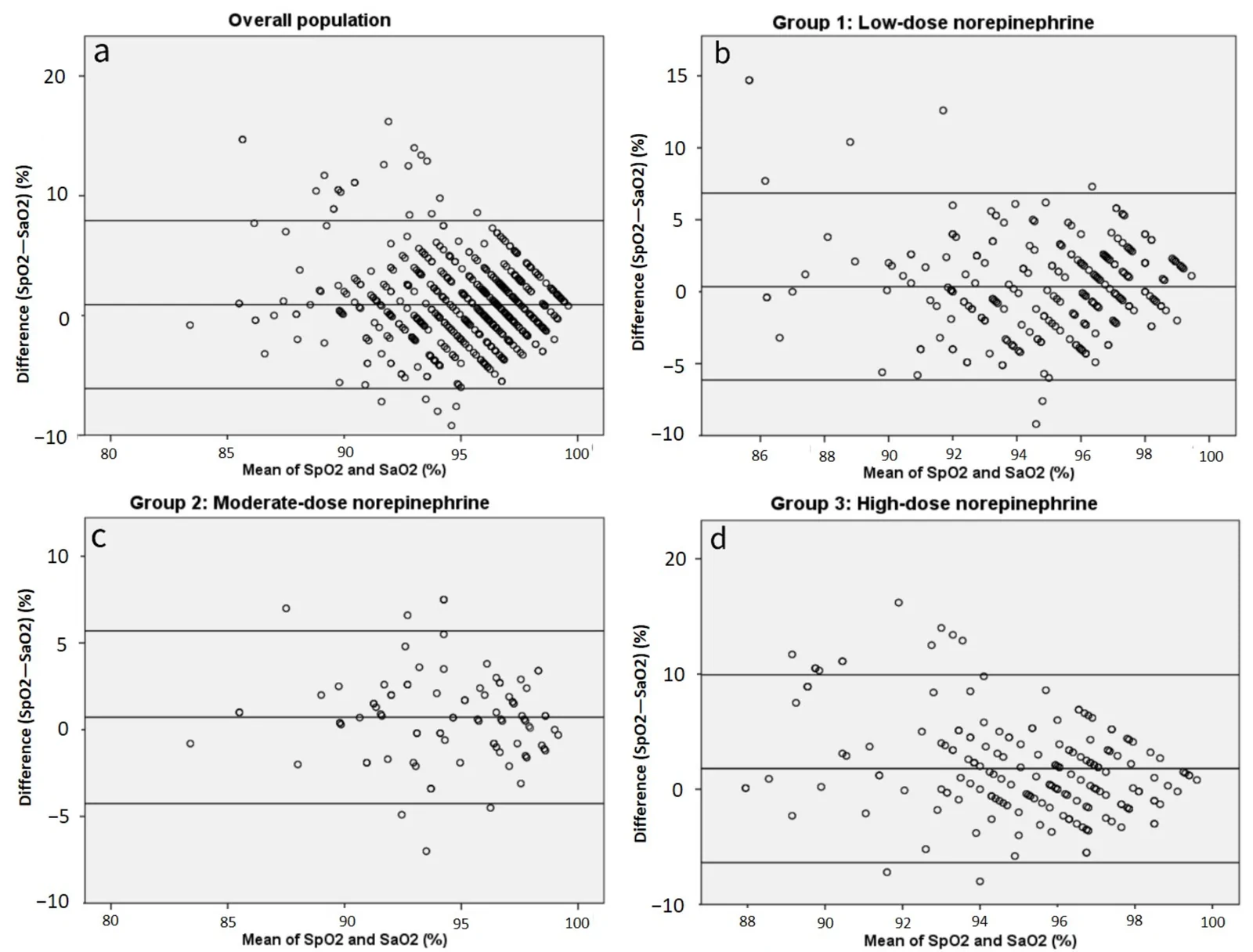
Descriptive, unadjusted density-based Bland–Altman plots illustrating SpO_2_–SaO_2_ agreement across norepinephrine dose groups. (**a**) Overall population; (**b**) Group 1: Low-dose norepinephrine; (**c**) Group 2: Moderate-dose norepinephrine; (**d**) Group 3: High-dose norepinephrine. The red dashed line represents the mean difference (bias), and the blue dotted lines represent the 95% limits of agreement.

In an exploratory analysis, linear regression demonstrated significant proportional bias between the SpO_2_–SaO_2_ difference and the mean saturation values (β = −0.198, *p* < 0.001; R^2^ = 0.025), indicating saturation-dependent measurement behavior (Figure 3).

In an exploratory analysis, age-stratified analyses (≥65 years vs. <65 years) revealed no clinically significant differences in the absolute SpO_2_–SaO_2_ discrepancy between age groups. Although statistically significant differences were observed, the effect size was small, suggesting limited clinical relevance.

In an exploratory descriptive analysis, we examined the distribution of discrepancy magnitudes across norepinephrine dose groups using fixed absolute thresholds (Supplementary Figure S3). Descriptively, higher norepinephrine doses appeared to be associated with a larger proportion of measurements in the ≥5% categories (21.5% in the high-dose group vs. 10.6% in the low-dose and 7.9% in the moderate-dose groups). However, because this analysis does not account for repeated measurements and no formal statistical testing was performed, these observations should not be interpreted as evidence of a dose-dependent effect. The primary GEE analysis, which adjusts for intra-patient correlation, did not confirm an independent association between norepinephrine dose and large discrepancies (≥5%).

## 4. Discussion

The principal finding of this study is that norepinephrine dose did not demonstrate a consistent dose–response relationship with large SpO_2_–SaO_2_ discrepancies after accounting for repeated measurements. Although one categorical comparison between the moderate- and high-dose groups reached statistical significance, the overall pattern was non-monotonic, confidence intervals were wide, and analyses treating norepinephrine dose as a continuous variable showed no statistically significant association. While our unadjusted descriptive data suggested greater variability at higher doses, these apparent differences may have been substantially influenced by within-patient variability and clustering rather than reflecting a consistent dose-dependent relationship.

Pulse oximetry is designed to operate within a limited accuracy range under conditions of stable peripheral perfusion, with typical performance targets approximating an ARMS of ~3% [7,11]. However, critically ill patients frequently deviate from these ideal conditions.

Our findings are consistent with prior studies demonstrating impaired pulse oximetry performance in low-perfusion states [12,13,14]. However, unlike previous work that used binary vasopressor classification (vasopressor yes/no), our study provides a dose-stratified evaluation. This is a key difference because dose stratification may better reflect the degree of vasopressor exposure than binary classification alone. Nevertheless, the absence of an independent association in the repeated-measures analysis suggests that norepinephrine dose alone is insufficient to explain pulse oximetry disagreement.

A notable finding was the dissociation between directional bias and measurement variability. Although the overall mean SpO_2_ overestimation was modest, individual measurements showed substantial variability, indicating that average bias alone may underestimate clinically relevant discrepancies. The observed dissociation between modest mean bias and substantial variability has important clinical implications: pulse oximetry error in this setting is not consistently directional but rather intermittent and unpredictable. This implies that clinicians cannot rely on a consistent “direction” of error; instead, an unpredictable and potentially large discrepancy may occur in any given measurement.

In descriptive Bland–Altman analyses, higher norepinephrine dose groups showed wider limits of agreement and greater dispersion (Figure 3), suggesting increased measurement variability across the saturation range. Although the proportional bias analysis revealed a statistically significant association between the SpO_2_–SaO_2_ difference and the mean saturation level (*p* < 0.001), the extremely low coefficient of determination (R^2^ = 0.025) indicates that the mean saturation explains less than 3% of the variance in measurement differences. This negligible effect size suggests that although saturation-dependent bias is statistically present, it is unlikely to be clinically meaningful in this dataset. The observed variability likely reflects multiple time-varying physiological and technical factors that cannot be explained by norepinephrine dose alone. The observed within-patient variability likely reflects time-varying physiological, technical, and clinical factors, including changes in peripheral perfusion, hemodynamic status, patient movement, sensor positioning, and evolving microcirculatory dysfunction. These findings mirror observations from contemporary ICU studies demonstrating that reduced pulse amplitude, low perfusion index, and impaired signal-to-noise ratio substantially degrade pulse oximeter accuracy during shock and vasopressor therapy [12,14,15].

Peripheral vasoconstriction induced by norepinephrine may contribute to impaired peripheral signal acquisition and has been associated with reduced agreement between invasive and noninvasive monitoring methods in previous studies [16]. These observations suggest a plausible physiological explanation for increased measurement variability during vasopressor therapy, although our primary repeated-measures analysis did not demonstrate an independent effect of norepinephrine dose.

Although larger discrepancies were observed descriptively in the high-dose group, this pattern was not supported in the primary GEE analysis. Therefore, norepinephrine dose alone should not be considered a reliable indicator of pulse oximetry performance. We do not recommend abandoning pulse oximetry in these patients; rather, we recommend using it as a screening tool with a lower threshold for confirmatory arterial blood gas sampling. Arterial blood gas analysis should be considered when SpO_2_ values are inconsistent with the clinical picture, particularly in patients with high FiO_2_ or suspected hypoxemia.

In our exploratory descriptive classification (Supplementary Figure S4), higher norepinephrine doses were descriptively associated with a larger proportion of measurements in the ≥5% categories. However, this pattern was not confirmed in the primary GEE analysis. Therefore, these exploratory observations should be interpreted solely as descriptive variability in measurements and should not be taken as evidence of a dose-dependent limitation in clinical monitoring. Although large discrepancies may occasionally yield falsely reassuring SpO_2_ values (so-called “occult” or “hidden” hypoxemia) [17,18], we emphasize that our classification is not a clinical risk-stratification tool and is not equivalent to saturation-dependent error-grid approaches [9]. In our cohort, this phenomenon was relatively infrequent, occurring in 36 of 1050 measurements (3.4%). However, when present, such discrepancies could lead to delayed recognition of hypoxemia and potentially affect clinical decision-making, particularly in patients with high oxygen requirements or those being weaned from ventilatory support.

The physiological mechanisms underlying these observations are likely multifactorial [19,20,21]. α-adrenergic–mediated peripheral vasoconstriction reduces capillary blood flow and pulse amplitude, both of which are fundamental determinants of pulse oximeter signal quality [13,19,20,22]. However, our descriptive findings are consistent with the possibility that these mechanisms contribute primarily to increased measurement variability rather than a predictable dose-dependent bias.

Several limitations should be acknowledged. First, our sampling strategy, which allowed measurements to be obtained ‘when clinically indicated,’ may have introduced selection bias. Episodes of clinical instability that prompted additional blood gas analyses could coincide with higher norepinephrine requirements and larger SpO_2_–SaO_2_ discrepancies. This could potentially exaggerate the descriptive association between norepinephrine dose and measurement error. Although the primary GEE analysis accounted for within-patient correlation and adjusted for measured covariates, residual confounding from unmeasured factors related to clinical instability and peripheral perfusion cannot be excluded. Furthermore, although we acknowledge that a sensitivity analysis restricted to fixed-interval measurements would have strengthened our findings, we were unable to perform such an analysis because the timing and clinical indication for each measurement were not systematically recorded in our dataset. This is a limitation of our real-world pragmatic design. Nonetheless, the consistency of our results across different analytical approaches (GEE, continuous dose analysis, and sensitivity analyses with alternative correlation structures) suggests that our main conclusions are robust. Second, although our model adjusted for measured covariates, residual confounding from unmeasured factors cannot be excluded. We acknowledge that norepinephrine dose may act as a marker of overall shock severity rather than an isolated determinant of pulse oximetry error. We did not record several factors that may influence pulse oximeter accuracy, including hemoglobin level, body temperature, peripheral perfusion index, capillary refill time, sensor site, and vasopressor duration. All of these could potentially confound the relationship between norepinephrine dose and SpO_2_–SaO_2_ agreement. We also did not systematically assess skin pigmentation, which is particularly relevant given recent FDA guidance highlighting racial and ethnic bias in pulse oximetry performance [3,17]. Emerging evidence suggests that pulse oximeters may overestimate arterial oxygen saturation in patients with darker skin pigmentation, potentially leading to occult hypoxemia. Although our study was not designed to evaluate this effect, and we did not record skin pigmentation data, this represents an important unmeasured confounder that could have influenced our results. Future studies specifically addressing the interaction between skin pigmentation, vasopressor therapy, and pulse oximeter accuracy are warranted. Consequently, our study is limited to evaluating the association of norepinephrine dose alone, and residual confounding from these unmeasured factors cannot be excluded. Third, our error classification used fixed absolute difference thresholds rather than saturation-dependent clinical risk zones. We acknowledge that the clinical significance of a 5% discrepancy is not uniform across the entire saturation range. For example, a 5% difference at an SpO_2_ of 95% (e.g., SpO_2_ 95% vs. SaO_2_ 90%) may have different clinical implications than a 5% difference at an SpO_2_ of 85% (e.g., SpO_2_ 85% vs. SaO_2_ 80%), where the oxygen–hemoglobin dissociation curve is steeper. Small changes in saturation reflect more substantial changes in partial pressure of oxygen. In our cohort, the majority of measurements were in the higher saturation range (median SaO_2_ 95.4%), and the overall frequency of large discrepancies was 13.9%. Nevertheless, we recognize that a fixed threshold approach does not capture the differential clinical risk at lower saturation levels. Future studies should consider saturation-dependent error grid analyses to characterize the clinical impact of SpO_2_ better–SaO_2_ discrepancies, particularly in patients with low baseline saturations or those requiring high FiO_2_. In patients with high FiO_2_ requirements, the clinical consequences of a large discrepancy may be particularly important. Although higher FiO_2_ was independently associated with reduced odds of large SpO_2_–SaO_2_ discrepancies in our cohort, when discrepancies do occur in this setting—especially at lower saturation levels—they may lead to inappropriate oxygen weaning or delays in recognizing hypoxemia. Clinicians should therefore maintain a low threshold for confirmatory arterial blood gas sampling in patients with high FiO_2_ requirements, even when SpO_2_ readings appear acceptable. Additionally, our exclusion of measurements obtained during concurrent administration of other vasoactive agents limits the generalizability of our findings. In clinical practice, refractory shock frequently requires combination vasopressor therapy (e.g., norepinephrine with vasopressin or epinephrine). Our results may not fully apply to patients receiving such combinations, as multiple vasoconstrictors could further impair peripheral perfusion and the accuracy of pulse oximetry. Therefore, our conclusions should be interpreted primarily for measurements obtained during norepinephrine monotherapy, and extrapolation to combination therapy should be made with caution. Finally, the observational design precludes causal inference, and residual confounding may persist despite our adjustment for measured covariates. Nevertheless, the prospective design, large number of paired measurements, and complementary analytical approaches strengthen the internal validity and clinical relevance of the findings. Additionally, the single-center design and the use of a single pulse oximeter platform (Philips IntelliVue MP20 with Masimo sensor) and blood gas analyzer (ABL 800 FLEX) limit the generalizability of our findings to other settings, devices, and patient populations. Different pulse oximeter devices and sensor technologies may exhibit variable performance characteristics, particularly under conditions of poor peripheral perfusion or vasoconstriction. The Masimo sensor used in our study incorporates signal-processing algorithms designed to improve performance in low-perfusion states; however, our findings may not generalize to other devices with different algorithms or sensor designs. Furthermore, the generalizability to other patient populations (e.g., different ICU settings, geographic regions) remains uncertain. Multi-center studies using diverse device platforms are needed to confirm the external validity of our findings.

Our findings have several practical implications for clinicians. First, although norepinephrine dose alone should not dictate the decision to obtain arterial blood gases, certain clinical scenarios may warrant confirmatory sampling. These include: (i) discrepancy between SpO_2_ and the clinical picture, (ii) increasing FiO_2_ requirements despite stable SpO_2_, (iii) hemodynamic instability or vasopressor escalation, and (iv) ambiguous or rapidly changing SpO_2_ trends. Second, although higher FiO_2_ was independently associated with lower odds of large discrepancies, this association should not be interpreted as improved device accuracy; it may reflect saturation-range effects, disease severity, or clinical management. The study did not evaluate a specific ABG-testing strategy or patient outcomes, and confirmatory sampling should remain guided by clinical judgment rather than by the FiO_2_ association alone. Third, when large discrepancies are suspected or when clinical decisions depend on accurate assessment of oxygenation (e.g., titration of supplemental oxygen, decisions regarding intubation or extubation), arterial blood gas analysis should be obtained rather than relying on SpO_2_ alone. Ultimately, SpO_2_ should be used as a screening tool. Still, its limitations in this population must be recognized, and confirmatory blood gas sampling should be guided by clinical judgment rather than by norepinephrine dose alone.

## 5. Conclusions

In this prospective repeated-measures study of critically ill patients receiving norepinephrine monotherapy, norepinephrine dose was not consistently associated with large SpO_2_–SaO_2_ discrepancies in the continuous-dose analysis. Although a non-monotonic categorical pattern was observed, the overall evidence does not support a consistent dose–response relationship. These findings indicate that norepinephrine infusion rate alone should not be used as a surrogate marker of pulse oximetry reliability or as the sole criterion for obtaining confirmatory arterial blood gas measurements. Rather, confirmatory testing should be guided by the overall clinical context, particularly when SpO_2_ values are inconsistent with the patient’s clinical condition or when oxygenation status is uncertain. Future studies incorporating direct measures of peripheral perfusion and standardized repeated-measurement protocols are needed to better identify the determinants of clinically relevant SpO_2_–SaO_2_ discrepancies.

## Figures and Tables

**Table 1:** Baseline demographic and clinical characteristics of the study population (*N* = 196 patients).

Variable	Median (IQR) or *n* (%)
**Demographic and anthropometric characteristics**	
**Age, years**	72 (59–78)
**Male sex**	126 (64.3)
**Body mass index, kg/m^2^**	24.8 (21.6–29.1)
**Disease severity and clinical conditions at ICU admission**	
**APACHE II score**	28 (23–37)
**Acute respiratory failure**	114 (58.2)
**Sepsis**	66 (33.7)
**Septic shock**	60 (30.6)
**Comorbidities**	
**Malignancy**	84 (42.9)
**Diabetes mellitus**	74 (37.8)
**Heart failure**	44 (22.4)
**Chronic kidney disease**	40 (20.4)
**Acute kidney injury**	48 (24.5)
**Cerebrovascular disease**	28 (14.3)
**Chronic obstructive pulmonary disease**	28 (14.3)
**Acute ischemic stroke**	6 (3.1)
**Other ICU admission diagnoses**	52 (26.5)

The most common diagnoses in the ‘other’ category included postoperative respiratory failure (*n* = 18, 9.2%), gastrointestinal bleeding (*n* = 10, 5.1%), trauma (*n* = 10, 5.1%), and acute pancreatitis (*n* = 8, 4.1%). Data are presented as median (interquartile range) or number (percentage), as appropriate. All variables in this table are summarized at the patient level (*N* = 196) to avoid bias related to repeated measurements. Disease severity scores and clinical diagnoses were recorded at the time of ICU admission. Sepsis and septic shock are reported separately to reflect differences in disease severity and vasopressor dependence.

**Table 2:** Multivariable GEE model for factors associated with large discrepancies (|SpO_2_ − SaO_2_| ≥ 5%).

Variable	Adjusted OR	95% CI	*p*-Value
**Norepinephrine dose group**			
**Low dose (≤0.25 µg/kg/min) vs. High dose (≥0.50)**	1.80	0.74–4.35	0.193
**Moderate dose (0.25–0.50 µg/kg/min) vs. High dose (≥0.50)**	3.49	1.17–10.44	0.025
**Norepinephrine dose (per 0.1 µg/kg/min increase)**	0.964	0.916–1.014	0.158
**Age (per year)**	1.012	0.993–1.030	0.219
**FiO_2_ (per 1 percentage-point increase)**	0.976	0.964–0.989	<0.001
**Lactate (per mmol/L)**	1.002	0.928–1.080	0.962
**Sepsis (yes vs. no)**	1.176	0.454–3.041	0.738
**Septic shock (yes vs. no)**	0.478	0.177–1.287	0.144
**Mechanical ventilation (yes vs. no)**	0.631	0.299–1.331	0.226

Dependent variable: Large_Discrepancy (1 = |SpO_2_ − SaO_2_| ≥ 5%, 0 = <5%). Reference category for norepinephrine dose: high-dose group (≥0.50 µg/kg/min). GEE with exchangeable working correlation structure, adjusted for within-patient clustering (*n* = 1050 measurements from 196 patients). Model fit: QIC = 845.016, QICC = 799.663. Analysis population: 1050 measurements from 196 patients; 146 measurements met the predefined large-discrepancy criterion (13.9%). Norepinephrine dose groups (measurements/patients, median dose [IQR]): low dose (≤0.25 µg/kg/min): 510 measurements/100 patients, median 0.13 (IQR 0.08–0.18) µg/kg/min; moderate dose (0.25–0.50 µg/kg/min): 178 measurements/36 patients, median 0.32 (IQR 0.28–0.40) µg/kg/min; high dose (≥0.50 µg/kg/min): 362 measurements/75 patients, median 1.19 (IQR 0.89–2.05) µg/kg/min. Sepsis and septic shock were coded as binary variables (yes/no).

## Data Availability

The datasets presented in this study are available from the corresponding author upon reasonable request, subject to institutional and ethical restrictions.

## Supplementary Materials

The following supporting information can be downloaded at: https://www.mdpi.com/article/10.3390/medicina62091687/s1, Supplementary Table S1. STROBE Statement checklist for observational studies. Supplementary Table S2. Measurement-level hemodynamic characteristics and SpO_2_–SaO_2_ discrepancies (*N* = 1050 paired measurements). Supplementary Table S3. Exploratory, unadjusted measurement-level comparisons of SpO_2_–SaO_2_ differences across norepinephrine dose groups (descriptive analysis only). Supplementary Table S4. Bland–Altman analysis using only the first measurement per patient (*n* = 196). Supplementary Figure S1. Bland–Altman plots using only the first measurement per patient (*n* = 196). Agreement between pulse oximetry (SpO_2_) and arterial oxygen saturation (SaO_2_) in (a) overall cohort, (b) low-dose norepinephrine (≤0.25 µg/kg/min), (c) moderate-dose (0.25–0.50 µg/kg/min), and (d) high-dose (≥0.50 µg/kg/min). Solid line: mean bias; dashed lines: 95% limits of agreement. This sensitivity analysis was performed to explore the impact of data reduction on descriptive agreement patterns and is secondary to the primary GEE analysis, which addresses a different question regarding dose–response association. The slight difference in bias between this sensitivity analysis and the primary analysis reflects the expected variability between using only the first measurement and using all measurements. Nevertheless, the descriptive pattern of wider limits of agreement in the higher norepinephrine dose groups remained consistent. Supplementary Figure S2. Receiver operating characteristic (ROC) curve of weight-adjusted norepinephrine dose for predicting large SpO_2_–SaO_2_ discrepancies (|SpO_2_ − SaO_2_| ≥ 5%). The area under the curve (AUC) was 0.60 (95% CI 0.55–0.65), indicating limited discriminatory ability. This exploratory analysis was performed to contextualize the primary GEE findings and was not intended to derive a clinical cutoff. Supplementary Figure S3. Exploratory descriptive classification of SpO_2_–SaO_2_ discrepancy magnitudes across norepinephrine dose groups. Panels show the distribution of absolute differences categorized as <2%, 2–5%, 5–10%, and >10% across low (≤0.25 µg/kg/min), moderate (0.25–0.50 µg/kg/min), and high (≥0.50 µg/kg/min) norepinephrine dose groups. This is an unadjusted, measurement-level descriptive analysis that does not account for repeated measurements. No formal statistical testing (e.g., trend analysis) was performed; the figure is intended solely to illustrate the dispersion of unadjusted data. The primary confirmatory analysis is the GEE model presented in the main text. This classification is not equivalent to saturation-dependent clinical risk assessment tools [9]. Note: Although this descriptive classification shows a higher proportion of large discrepancies in the high-dose group, this trend was not statistically significant in the primary GEE analysis after accounting for repeated measurements. Therefore, no dose-dependent independent association should be inferred from this figure.

## Abbreviations

The following abbreviations are used in this manuscript:

ABG	Arterial blood gas
APACHE II	Acute Physiology and Chronic Health Evaluation II
ARMS	Accuracy root mean square
AUC	Area under the curve
BMI	Body mass index
CI	Confidence interval
COPD	Chronic obstructive pulmonary disease
FDA	U.S. Food and Drug Administration
FiO_2_	Fraction of inspired oxygen
GEE	Generalized estimating equation
ICU	Intensive care unit
IQR	Interquartile range
ISO	International Organization for Standardization
LoA	Limits of agreement
MV	Mechanical ventilation
OR	Odds ratio
QIC	Quasi-likelihood under the independence model criterion
ROC	Receiver operating characteristic
SaO_2_	Arterial oxygen saturation measured by co-oximetry
SpO_2_	Peripheral oxygen saturation measured by pulse oximetry
STROBE	Strengthening the Reporting of Observational Studies in Epidemiology
VIF	Variance inflation factor
