# Supplementary material for: Norepinephrine Dose and Pulse Oximetry Agreement During Critical Illness: A Prospective Repeated-Measures Study
Source: Medicina (Kaunas). 2026 Sep 3;62(9):1687. doi: 10.3390/medicina62091687 (PMC13608892; doi:10.3390/medicina62091687)
Supplement: Supplementary file 1 [file medicina-62-01687-s001.zip › Supplementary Table S2.pdf]

Supplementary Table 2. Measurement-level hemodynamic characteristics and SpO_2–SaO_2 discrepancies (N = 1,050 paired measurements)

Variable	Median (IQR) or n (%)
**Hemodynamic and respiratory parameters at the time of measurement**	
Weight-adjusted norepinephrine dose, µg/kg/min	0.27 (0.13–0.89)
Low-dose norepinephrine (≤0.25 µg/kg/min)	510 (48.6)
Moderate-dose norepinephrine (0.25–0.50 µg/kg/min)	178 (17.0)
High-dose norepinephrine (≥0.50 µg/kg/min)	362 (34.5)
Serum lactate, mmol/L	3.4 (1.9–6.7)
Fraction of inspired oxygen (FiO_2), %	40 (29–60)
Invasive mechanical ventilation at measurement	820 (78.1)
**Oxygenation and pulse oximetry measurements**	
Arterial oxygen saturation (SaO_2), %	95.4 (92.7–97.0)
Simultaneous pulse oximetry saturation (SpO_2), %	96 (93.8–98)
Signed difference ($SpO_2 - SaO_2$), %	0.6 (−1.2 to 2.6)
Absolute difference $\lvert SpO_2 - SaO_2 \rvert$, %	2.0 (1.0–3.7)
Clinically meaningful pulse oximetry error (≥5%)	146 (13.9)

Data are presented as median (interquartile range) or number (percentage), as appropriate. All variables are summarized at the measurement level (N = 1,050 paired SpO_2–SaO_2 measurements). Percentages may not total 100% due to rounding. Mechanical ventilation status, FiO_2, and norepinephrine dose reflect conditions at the time of each paired measurement. Clinically meaningful pulse oximetry error was defined as an absolute SpO_2–SaO_2 difference ≥5%. Serum lactate reflects perfusion status at the time of measurement and was included as a prespecified severity covariate.
