# Supplementary material for: Norepinephrine Dose and Pulse Oximetry Agreement During Critical Illness: A Prospective Repeated-Measures Study
Source: Medicina (Kaunas). 2026 Sep 3;62(9):1687. doi: 10.3390/medicina62091687 (PMC13608892; doi:10.3390/medicina62091687)
Supplement: Supplementary file 1 [file medicina-62-01687-s001.zip › Supplementary Table S3.pdf]

Supplementary Table S3. Exploratory, unadjusted measurement-level comparisons of SpO_2–SaO_2 differences across norepinephrine dose groups (descriptive analysis only)

Norepinephrine dose group (µg/kg/min)	Measurements (N)	Signed difference ($SpO_2 - SaO_2$), median (IQR)	Absolute difference $\|SpO_2 - SaO_2\|$, median (IQR)
**Low (≤0.25)**	510	0.10 (−1.80 to 2.20)	2.0 (1.0 to 3.7)
**Moderate (0.25–0.50)**	178	0.70 (−0.80 to 2.10)	1.8 (0.9 to 3.1)
**High (≥0.50)**	362	1.20 (−0.90 to 3.70)	2.7 (1.3 to 4.4)

Overall comparisons

- **Signed difference:** Kruskal–Wallis $\chi^2(2) = 24.77$, $p < 0.001$
- **Absolute difference:** Kruskal–Wallis $\chi^2(2) = 18.57$, $p < 0.001$

Note: These are unadjusted comparisons that treat each measurement as independent. They do not account for repeated measurements within the same patient. The primary GEE analysis, which adjusts for within-patient correlation, is the confirmatory analysis (see Table 2 and text for adjusted odds ratios). These descriptive findings are exploratory and should not be interpreted as confirmatory evidence or used for causal inference.
