# Supplementary material for: Norepinephrine Dose and Pulse Oximetry Agreement During Critical Illness: A Prospective Repeated-Measures Study
Source: Medicina (Kaunas). 2026 Sep 3;62(9):1687. doi: 10.3390/medicina62091687 (PMC13608892; doi:10.3390/medicina62091687)
Supplement: Supplementary file 1 [file medicina-62-01687-s001.zip › Supplementary Table S4.pdf]

Supplementary Table S4. Bland–Altman analysis using only the first measurement per patient (n = 196)

Norepinephrine dose group	n (patients)	Bias (%)	Lower LoA (%)	Upper LoA (%)	LoA width (%)
**Overall**	196	–0.76	–7.57	+6.05	13.62
**Low (≤0.25 µg/kg/min)**	108	+0.11	–6.17	+6.39	12.56
**Moderate (0.25–0.50)**	30	–0.71	–6.75	+5.33	12.08
**High (≥0.50)**	58	–2.39	–9.49	+4.71	14.20

LoA: limits of agreement (mean bias ± 1.96 × SD)
